# Iron Oxide Nanoparticles: Green Synthesis and Their Antimicrobial Activity

**DOI:** 10.3390/nano13222919

**Published:** 2023-11-08

**Authors:** Johana Zúñiga-Miranda, Julio Guerra, Alexander Mueller, Arianna Mayorga-Ramos, Saskya E. Carrera-Pacheco, Carlos Barba-Ostria, Jorge Heredia-Moya, Linda P. Guamán

**Affiliations:** 1 Centro de Investigación Biomédica (CENBIO), Facultad de Ciencias de la Salud Eugenio Espejo, Universidad UTE, Quito 170527, Ecuador; johana.zuniga@ute.edu.ec (J.Z.-M.); arianna.mayorga@ute.edu.ec (A.M.-R.); saskya.carrera@ute.edu.ec (S.E.C.-P.); jorgeh.heredia@ute.edu.ec (J.H.-M.); 2 Facultad de Ingeniería en Ciencias Aplicadas, Universidad Técnica del Norte, Ibarra 100107, Ecuador; jeguerra@utn.edu.ec; 3 Department of Molecular Biology, Princeton University, Princeton, NJ 08544, USA; amm9@princeton.edu; 4 Escuela de Medicina, Colegio de Ciencias de la Salud Quito, Universidad San Francisco de Quito USFQ, Quito 170901, Ecuador; cbarbao@usfq.edu.ec; 5 Instituto de Microbiología, Universidad San Francisco de Quito USFQ, Quito 170901, Ecuador

**Keywords:** antimicrobial resistance, green synthesis, IONPs, antibacterial activity, antifungal activity, antiparasitic, antiviral activity

## Abstract

The rise of antimicrobial resistance caused by inappropriate use of these agents in various settings has become a global health threat. Nanotechnology offers the potential for the synthesis of nanoparticles (NPs) with antimicrobial activity, such as iron oxide nanoparticles (IONPs). The use of IONPs is a promising way to overcome antimicrobial resistance or pathogenicity because of their ability to interact with several biological molecules and to inhibit microbial growth. In this review, we outline the pivotal findings over the past decade concerning methods for the green synthesis of IONPs using bacteria, fungi, plants, and organic waste. Subsequently, we delve into the primary challenges encountered in green synthesis utilizing diverse organisms and organic materials. Furthermore, we compile the most common methods employed for the characterization of these IONPs. To conclude, we highlight the applications of these IONPs as promising antibacterial, antifungal, antiparasitic, and antiviral agents.

## 1. Introduction

Microbial agents such as fungi, bacteria, parasites, and viruses cause various infectious diseases and can be treated with antimicrobial agents. The main concern in treating infectious diseases is that many pathogenic microorganisms have developed resistance against antimicrobial agents, for example, antibiotics [1]. The development of antibiotic resistance is the result of the inappropriate use of antibiotics in community, clinical, and agricultural settings. Consequently, multidrug-resistant bacteria have become a global health threat [2]. Therefore, there is an urgent need to find new and effective agents to overcome drug resistance. In this regard, nanotechnology reveals new opportunities for the synthesis of nanoparticles with antibacterial properties to solve these challenges [3]. The field of nanoparticles has gained significant attention in recent decades due to their unique properties, diverse synthesis methods, and wide-ranging applications. The synthesis of nanoparticles involves various techniques, including chemical, physical, and biological methods (green synthesis) [4]. Chemical and physical methods, such as chemical reduction [5] and electrodeposition [6], produce nanoparticles with controlled size, shape, and composition. However, some chemical and physical synthesis methods often involve high energy consumption, the generation of hazardous by-products, and use of toxic chemicals, which can adversely affect biological systems [4]. In contrast, green synthesis methods that use natural extracts [7,8] offer environmentally friendly and sustainable approaches to nanoparticle synthesis. They eliminate or minimize the use of toxic chemicals and reduce energy consumption [9,10]. Additionally, they offer a one-step process without additional chemical coatings, simplifying the synthesis process [4]. Green synthesized nanoparticles exhibit enhanced biocompatibility and reduced toxicity compared to chemically synthesized nanoparticles [11,12].

The metallic nanoparticles are made through a bottom-up approach that includes a chemical reduction in which a natural extract containing compounds such as polyphenols acting as reducing and stabilizing agents during the synthesis process [13] replaces a toxic chemical-reducing agent. It is essential to identify the biomolecules present in the natural extract since this would allow us to understand the role that the compounds in the extract play in the nucleation, growth, and stabilization of the nanoparticles [14]. The different compounds in the extract can exert different functions in the synthesis by acting as reducing, protective, and stabilizing agents, and they can even provide specific functionality to the nanoparticles [15,16,17]. Therefore, identifying these components helps optimize synthesis conditions and leads to the development of nanoparticles with specific characteristics and applications [18,19]. Furthermore, knowing the main components of an extract ensures that the synthesis process can be accurately reproduced in different settings [14]. A consistent composition leads to consistent nanoparticle properties [20,21]. In biomedical applications, by knowing the components of the extract, contaminants or byproducts that may affect the safety profile of the nanoparticles can be determined [22]. 

Iron oxide nanoparticles (IONPs) are defined as “Nanozymes” due to their inherent enzyme-like activities and catalytic properties that are comparable to those of different oxidases such as peroxidases, catalase, sulfite oxidase, and superoxide dismutase [23]. Another feature of these particles is their potential to bind different biological molecules like peptides, enzymes, nucleic acids, lipids, fatty acids, and various metabolites. These nanoparticles have applications in optics, mechanics, biotechnology, engineering, remediation, microbiology, environmental medicine, and electronics, among others [19,24]. Research on the green synthesis of iron oxide nanoparticles and their antimicrobial activity has grown rapidly and gained notoriety in the last 10 years. Highlighting the relevance of this research area in recent years, according to information obtained from the Web of Science database, in the last decade (2013–2023), 335 articles were published with the keywords (“Iron Oxide Nanoparticles” OR “Fe_3_O_4_ nanoparticles” OR “magnetite nanoparticles”) AND (“Green Synthesis” OR “sustainable synthesis” OR “eco-friendly synthesis”) AND (“Antimicrobial Activity” OR “antibacterial” OR “antifungal” OR “antimicrobial properties”), compared to 1 article in the previous decade (2002–2012) using the same search terms. This represents a growth of 33500% (335 times the number of articles) in the last 10 years. This review focuses on green methods for synthesizing iron oxide nanoparticles using bacteria, fungi, yeasts, plant extract, and organic waste. In addition, IONP’s prospective antimicrobial applications are discussed in detail. This review will provide compelling information for researchers in the field of nanobiotechnology.

## 2. Green Synthesis of Iron Oxide Nanoparticles

### 2.1. Synthesis of Iron Oxide Nanoparticles Using Microorganisms

The synthesis of iron oxide nanoparticles (IONPs) using microorganisms has several advantages over other methods due to the relative abundance of microorganisms, the generation of less toxic by-products for biomedical purposes, and lower cost [25]. Several microorganisms have the ability to accumulate and detoxify metals, and this property can be exploited to convert iron ions from metal salts into nanoparticles [26] using a variety of enzymes, cofactors, proteins, and secondary metabolites of the microorganism [26,27]. These molecules can act not only as reducing agents but also play an important role as stabilizers and protectors of nanoparticles [26]. Bacterial proteins containing functional groups, such as hydroxyl, thiol, carboxylic acid, and amine_,_ have been demonstrated to play a role in the stabilization of metal nanoparticles. These functional groups serve as binding sites for metal ions, which are subsequently reduced to form nanoparticles within the cell wall or the periplasmic space [28].

The synthesis of nanoparticles using microorganisms such as bacteria, fungi, yeasts, and algae can be carried out by intracellular or extracellular mechanisms [27]. The extracellular mechanism consists of the reduction of metal ions, such as iron, by proteins, enzymes, or components of the cell wall of microorganisms [29]. These components work as reducing agents and stabilizers and prevent the agglomeration of iron oxide nanoparticles [28]. For this type of synthesis, the microorganisms are grown under optimal conditions, the supernatant is collected by centrifugation and mixed with an aqueous solution of the metal salt, and the presence of nanoparticles is evidenced by the change in color of the medium [26]. Metal nanoparticles can also be formed when metal ions are trapped on the cell surface by electrostatic interaction and are reduced by enzyme action [28].

**Figure 1:**
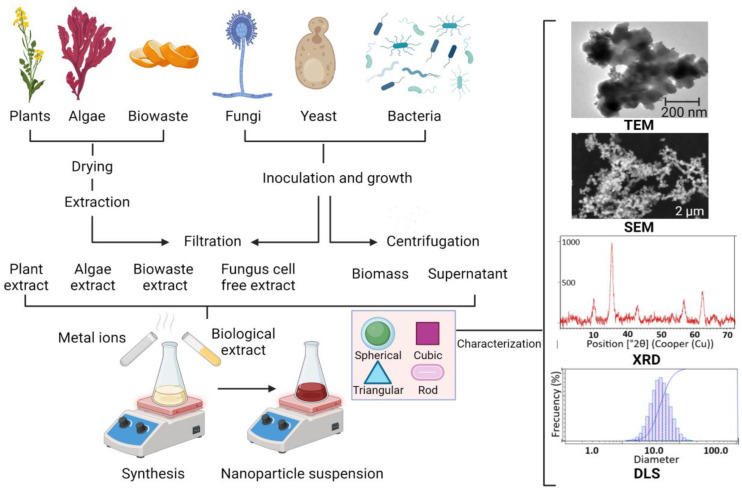
Schematic representation of green synthesis of iron oxide nanoparticles. Figure created with BioRender.com.

Intracellular mechanisms involve the enzymatic reduction of metal oxide ions through the electrostatic binding of metal ions to -COOH groups of the microbial cell wall, metal ions diffuse into the cell and interact with enzymes to form metal oxide nanoparticles [30,31]. For this type of synthesis, the microorganisms are grown under optimal conditions, then the biomass is washed with sterile water and incubated with a solution of metal salts until a color change indicates the nanoparticle synthesis [26] (Figure 1).

The reduction of metal ions is under the control of several factors, such as functional groups in the cell wall, type of strain, pH, temperature, culture medium, and metal salt concentration. These factors directly or indirectly affect the size, shape, composition, and performance of nanoparticles [32].

Purifying nanoparticles after the synthesis is crucial to remove impurities and ensure the quality of the nanoparticles. Methods such as centrifugation help separate the reaction mixture’s nanoparticles [33,34]. Another purification method is filtration; filters of different pore sizes can be used to remove larger particles and aggregates from the nanoparticle suspension [35,36]. Dialysis is another method that separates nanoparticles from small molecules and ions through a semipermeable membrane [37]. Although the methods described are commonly used, other techniques such as chromatography, electrophoresis, or flocculation are also used to purify nanoparticles [38]. The selection of the purification method should be based on the specific characteristics of the nanoparticles and contaminants in the suspension.

### 2.2. Synthesis of Iron Oxide Nanoparticles Using Bacteria

The synthesis of iron oxide nanoparticles generally begins with the selection and isolation of bacteria; this includes culturing the strain on specific media such as BHI agar or nutrient agar, followed by incubation under controlled conditions at 35–37 °C for 24–48 h. Additionally, conditions such as carbon dioxide atmosphere may be required. The obtained individual colonies are transferred to the nutrient medium, such as Luria Bertani or nutrient broth, followed by incubation and centrifugation [39,40]. The supernatant (extracellular method) or the cell pellet (intracellular method) should be obtained depending on the synthesis mechanism. The supernatant or sediment suspension is combined with the precursor salts, such as FeCl_3_·6H_2_O or Fe_2_O_3_, in the desired volume and concentration. The reaction conditions are optimized by adjusting the pH to the preferred range (5–9), and the mixture is then incubated at different temperatures depending on the strain of bacteria used. After incubation, the color of the medium changes, for example, from dark red to dark brown, indicating the production of IONP nanoparticles [41]. When using the intracellular approach, an additional step is required to remove the nanoparticles from inside the cell; for this, a heat shock must be performed by resuspending the cells in buffer, centrifuging, and then using liquid nitrogen followed by a steam bath at 37 °C. The supernatant is centrifuged and collected as a cytoplasmic extract [26,42]. Finally, the synthesized IONPs are dried and characterized. The synthesis of IONPs via different bacterial species is summarized in Table 1.

### 2.3. Synthesis of Iron Oxide Nanoparticles Using Fungi

The production of iron oxide nanoparticles via fungal synthesis is considered an attractive option due to its simplicity of scaling, the low cost of the materials necessary for growth, the capacity to produce high amounts of biomass even at an industrial scale, and the low toxicity of the residue [58,59]. In addition, fungal species have higher metal tolerance and bioaccumulation properties, facilitating the synthesis of metal nanoparticles [60]. Fungi are known to secrete large amounts of proteins, enzymes (i.e., reductase enzymes [33,61] which could be involved in converting metal salts into various metal nanoparticles [30,62]. Researchers discovered that chitin, a key component of the fungal wall system, is involved in the formation of heavy metal complexes, which are linked to the synthesis of nanoparticles [63]. Other studies suggest that the presence of functional groups in biomolecules of fungus such as carboxylic acid, amide, and alkyl and phenolic compounds could be involved in the capping and reducing process [64,65]. However, the mechanisms behind metal nanoparticle synthesis using fungi still need to be fully understood. 

To optimize the synthesis, some factors must be considered, including the microorganism strain, growth parameters, sample preparation technique, metallic precursor, metallic salt concentration, pH, and reaction temperature [66]. Metal nanoparticles may be synthesized using fungi in a variety of ways, including employing fungal biomass, cell-free filtrate (CFF) from fungal culture, or fungal cell filtrate (FCF) [27]. The use of mycelium is one of the simplest approaches; this process involves culturing the selected fungus strain in an appropriate medium and then separating the mycelium by centrifugation. The mycelium is combined with a metal precursor salt solution and incubated for 4–5 days at 28 °C. The solution is then filtered to produce a colloidal suspension containing the synthesized nanoparticles [67,68]. Nanoparticles can also be synthesized using the cell-free filtrate, which is obtained by growing fungi in a suitable medium and separating the mycelium by filtration. After washing and resuspending the fungal biomass in distilled water, it is incubated on a rotary shaker for 1–5 days, and the filtrate is collected by separating the mycelium with filter paper. Finally, the cell-free filtrate is combined with a solution of iron precursor salts such as ferric chloride and incubated for a few hours on the rotary shaker to achieve IONP synthesis [62]. For the IONP synthesis method based on fungal cell filtrate, the fungal strain is cultivated in the appropriate medium, after which the culture medium is filtered, and the filtrate obtained is centrifuged and filtered again to obtain the final FFC. Finally, a FeCl_3_ solution is stirred at room temperature for 5 min. An immediate visual change in color reveals IONP synthesis [69].

On the other hand, relatively little is known about IONP biosynthesis from yeast. This type of fungus has a widely varied metabolic and proteomic profile, which means it can produce a wide variety of compounds that have the potential to act as reducing agents [70]. However, more research is required to evaluate its potential and biosynthetic mechanism. Recent reports on the synthesis of IONPs by yeast and fungi are shown in Table 2.

### 2.4. Synthesis of Iron Oxide Nanoparticles Using Algae

Algae (microalgae and macroalgae) are commonly used to produce different metallic nanoparticles, and this has become an environmentally friendly option because it is a clean, non-toxic, and sustainable process with the potential to produce a wide variety of sizes, shapes, compositions, and physical and chemical properties [76]. Glycoproteins and different functional groups, such as carbonyl, hydroxyl, and carboxyl, as well as proteins, enzymes, alkaloids, carotenoids, vitamins, terpenes, and cell wall polysaccharides [77,78], are just a few of the biomolecules found in algae. Furthermore, algae can also accumulate in heavy metals and produce secondary metabolites with anti-biological fouling effects [76,79]. These characteristics, together with the components they produce, are critical in the reduction, protection, manufacture, and stabilization [27] of nanoparticles [80]. For example, an aqueous extract of *Sargassum muticum* was used to synthesize cubic iron oxide nanoparticles from FeCl_3_ at a temperature of 25 °C, and it was determined that the polysaccharides in the algae extract were the main factor in the reduction of FeCl_3_ to Fe_3_O_4_ nanoparticles with an average size of 18 ± 4 nm [78].

To synthesize nanoparticles from algae, the samples are washed with distilled water and then dried for 72 h under light [78]. The dried samples are then powdered, freeze-dried, weighed, and boiled by mixing the powder with the necessary volume of distilled water. To produce the extract, the boiled extract is subjected to a filtration step. The synthesis of the IONPs begins by combining the extract with a known amount of iron salts, such as FeCl_3_ [78,81]. The color change in the media indicates that nanoparticle nucleation has occurred [76,82]. This process is facilitated by the biomolecules found in algae extract, which facilitate the aggregation and self-assembly of nucleonic particles. During this process, the most energetically favorable and stable particle shapes are generated, with cubes, hexagons, pentagons, bars, spheres, triangles, and wires being the most prevalent [83]. Table 3 summarizes information on nanoparticles obtained from various algae extracts.

### 2.5. Synthesis of Iron Oxide Nanoparticles Using Plants

Because microorganism-based synthesis requires long incubation periods, extensive research has been conducted on the plant-based synthesis of iron oxide nanoparticles since plants, on the other hand, reduce metal salts in a shorter time [26]. Plant extracts used for the biosynthesis of nanoparticles can be obtained from the leaves, flowers, bark, roots, fruits, and stems [27]. Bioactive compounds of these extracts, including mainly tannins, phenols, alkaloids, saponins, organic acids, flavonoids, and vitamins, work as reducing agents for metal salts and stabilizers of nanoparticles [7,27]. 

Depending on the iron precursor used to form the Fe^0^ metal, electron-rich biomolecules containing hydroxyl groups (-OH) have the ability to effectively reduce iron ions from the divalent or trivalent oxidation state (Fe^2+^ or Fe^3+^) [94,95]. The characteristics of the synthesized nanoparticles are mostly determined by discrete parameters such as plant extract type, volume ratio of the extract, metal salt solutions, and reaction variables such as pH, temperature, and incubation time [94,96].

To obtain the plant extract, the first step is to collect the desired part of the plant of interest, which is then washed with distilled water to remove any contaminants, dried at room temperature, and then ground into a fine powder to provide a good surface for extraction [95]. The active compounds of this powder are extracted by mixing it with the appropriate solvent, either hot or cold; the most frequently employed solvents are water, methanol, ethanol, or aqueous dilutions of these [97,98]. Finally, it is filtered, and the clear solution is mixed with the iron precursor salts (iron chloride, iron nitrate, or iron sulfate) to produce IONPs. In this case, the chemical components in the extract also stabilize the nanoparticles, and the change in color of the solution, which depends on the kind of salt used, intensifies as the nanoparticles are synthesized [24,95,99]. As shown in Table 4, various parts of plants have been studied for the environmentally safe synthesis of iron oxide nanoparticles.

### 2.6. Green Synthesis of Iron Oxide Nanoparticles Using from Biological Waste Products

Agro-wastes are abundantly produced during the processing of agricultural products and are frequently discarded into the environment, causing pollution [140]. Agro-waste has been found to be rich in biomolecules such as flavonoids in peel fruits [141], polysaccharides, caffeine and tannic acid in tea waste [142], and proteins [143] that can serve as bioreductant agents in the green synthesis of diverse metallic nanoparticles [144]. A new effort to expand the use of agro-wastes as sources of biomolecules in green nanotechnology has received considerable attention, and various agro-wastes have been documented for their relevance in nanobiotechnology [145]. As a result, a range of metallic nanoparticles have been successfully synthesized from agro-industrial wastes, and extracts from these residues have been employed as reducing and stabilizing agents in the majority of cases [146]. The nanoparticles produced in this way have a wide range of activities, including antimicrobial, antioxidant, anticorrosive, and cytotoxic against cancer cells, among others [146,147,148,149]. In general, extracts containing active biomolecules that promote the formation of nanoparticles can be prepared using a simple hot water extraction process of dried and ground agro-waste materials [145]. Information about the nanoparticles obtained from biological waste is highlighted in Table 5.

## 3. Characterization Methods

The green synthesis methods of metallic nanoparticles are continually being improved and optimized to achieve comparable results (size and morphology) to physical and chemical methods. Usually, a change in the color of the reaction mixture indicates the formation of nanoparticles; however, this does not guarantee their formation, so additional processes must be used to identify the particles formed. Continuous UV spectra of the reaction mixture are one of the most frequently applied methods to confirm the formation of nanoparticles [119,162]. Currently, a variety of characterization methods are useful for evaluating the properties and uses of synthesized nanoparticles, as well as optimizing synthesis procedures [163]. However, no single instrument can characterize all material properties. As a result, numerous complementary and confirmatory techniques for nanoparticle characterization should be applied (Table 6).

The most common nanoparticle parameters studied include size, shape, charge, and surface topology [163]. However, characterizing nanoparticles remains difficult due to the sample preparation procedure, and the characterization technique utilized is directly dependent on the properties required [164].

One of the most important parameters to evaluate is particle size and size distribution, which correlates to the particle’s external dimensions. Because this property can determine other properties and the behavior of the final product [165,166], it is important for biological and biomedical applications. This property frequently differs depending on the method employed to evaluate it, which can be attributable to a variety of factors. For example, different methods evaluate different aspects of nanoparticle dimensions, or the method can induce a change in the effective particle size [167]. Among the most common methods for determining the size of iron oxide nanoparticles are transmission electron microscopy (TEM) [101,149,168,169], scanning electron microscopy (SEM) [7,149,170,171], X-ray diffraction (XRD) [170,172,173,174], dynamic light scattering (DLS) [175,176,177], high-resolution transmission electron microscopy (HRTEM) [178,179,180], and scanning transmission electron microscopy (STEM) [119].

The physical appearance of a particle (morphology), which comprises shape and surface topography, is important to the behavior of iron oxide nanoparticles. Morphology affects different properties like dispersion, functionality, and toxicity. This property, like the size, is evaluated through direct viewing of the particles, so commonly, electron microscopy techniques are utilized. Among the various forms of iron oxide nanoparticles that have been reported are round (SEM) [181], spherical (SEM) [170,171], (STEM) [119], (TEM) [174], spheroidal (HRTEM) [180], cubic (SEM) [171], rectangular and triangular (HRTEM) [179], rhombohedral [149], and needle-like (FESEM and HRTEM) forms [182].

Another important property to determine is the chemical composition of iron oxide nanoparticles. It can be characterized both in terms of surface and bulk chemistry and can be measured at the ensemble or single nanoparticle level. It is mostly determined using spectroscopic techniques, which may be paired with microscopy to attain single-particle-level resolution, with the most often used methods being XRD [101,180,183,184], EDS [11,119,183], and EDX [172,185,186,187]. Because the nanoparticle’s external layer interacts with the surrounding environment, determining its composition (surface chemistry) is also important and can be obtained using different techniques like Auger electron spectroscopy [188,189], X-ray photoelectron spectroscopy [190,191,192], or inductively coupled plasma mass spectrometry [193].

Along with the chemical composition, another property of importance in the study of IONPs is the crystalline structure of the nanoparticles. In general, XRD is used to determine the three-dimensional arrangement of the atoms in nanoparticles as well as the different phases. However, it has been found that nanoparticles smaller than 5 nm have an impact on the XRD-observed patterns [194]. SAED [195], which is increasingly commonly being used, is a method for a more accurate representation of the crystalline structure of iron oxide nanoparticles [174,177,183].

The charge of nanoparticles in suspension is also an important property to consider. The surface charge is the charge formed on the surface of the nanoparticle as a result of proton adsorption or desorption at hydroxylated sites, and due to the difficulties of directly measuring this charge, the particle’s zeta potential is frequently determined. The zeta potential (ζ) is the difference in potential between the stationary layer of the dispersion medium attached to the dispersed particle and the mobile dispersion medium [195] and can be measured by titration [196] or electrophoresis [119,197,198]. It is important because it is used as an indicator of colloidal stability, which is a critical parameter for many emulsions or dispersions. Highly charged particles have high zeta potentials and repel each other, resulting in stable colloidal solutions with few agglomeration trends. These highly charged particles are associated with pH levels far from the “isoelectric point”, or the pH value at which the zeta potential is zero. On the other hand, low zeta potential values cause colloids to flocculate, corresponding to values closer to the system’s isoelectric point. Colloids with zeta potential values of 20–30 mV or above are considered stable [164]. In general, a decrease in zeta potential is observed as pH increases [199,200]. The zeta potential has been used to investigate the interaction of iron oxide nanoparticles with various biological systems, including human cells [201], bacteria [202], and proteins [203].

Stabilizing IONPs is critical for generating suspensions that resist aggregation in biological environments and magnetic fields. Obtaining particle stability requires a balance of attracting and repulsive forces, which may be generated via electrostatic and steric repulsion. The efficacy of these forces is critical for ensuring high particle stability. The steric force is difficult to predict and measure. However, electrostatic repulsion may be monitored using the diffusion potential, which is often very close to the zeta potential. As mentioned above, the solution’s ionic strength and pH heavily influence the diffusion potential [204]. Therefore, modifying these parameters and others, such as temperature, surface coating of IONP, or concentration variations, can substantially impact the IONP stability [197].

Surface iron atoms in IONPs act as Lewis acids, coordinating with molecules with lone–pair electrons. When Fe atoms interact with water in aqueous solutions, they dissociate, and the surface becomes hydroxyl functionalized. Due to the amphoteric character of these hydroxyls, the nanoparticles may react with both acids and bases, and the IONP’s surface can be positive or negative, depending on the pH of the solution. The isoelectric point is at pH 6.8; around this point, the surface charge density is too low, causing the particles to flocculate [197,204]. It is interesting to note that adding an acetate buffer (pH = 3) can stabilize and prevent the aggregation of amorphous IONP produced from an *H. vulgare* plant extract (pH = 5.8). These nanoparticles are unstable by nature and prone to aggregation. A similar effect was observed when *R. acetosa* extracts were used to produce IONPs. These extracts, however, vary from those of *H. vulgare* primarily in their acidic pH (pH = 3.7). Organic acids, such as oxalic or citric acids, are essential for iron nanoparticle stability, according to the authors of this study, suggesting that plants containing these compounds may be more effective for green IONP synthesis [96].

Nanoparticles’ magnetic behavior differs from that of bulk materials, as do other properties. Furthermore, the synthesis method, size, and shape of IONPs all affect the magnetic properties of the nanoparticles [205]. When the particle size is between nano and micro, it exhibits superparamagnetism, resulting in low remanent magnetization and a coercive field. As a result, the nanoparticles can interact with an external magnetic field, but in the absence of this field, the particles exhibit no magnetism. 

The superparamagnetic behavior of nanoparticles improves as their size decreases, whereas the ferromagnetic behavior decreases [206]. Magnetic Fe_3_O_4_ nanoparticles, for example, are superparamagnetic below the size of 20 nm [207]. Furthermore, it has been observed that the magnetic properties of iron nanoparticles vary depending on the temperature at which they are measured and the degree of order of magnetic dipoles [208,209]. This is mainly because high temperatures can produce changes in bond lengths, angles, and coordination numbers at nanostructure surfaces [210]. Increased temperatures cause an increase in thermal energy, which facilitates dipole alignment and increases magnetism up to the blocking temperature. However, when the temperature increases, magnetization decreases due to reduced dipole alignment with the field [210,211].

Because of this property, superparamagnetic iron oxide nanoparticles (SPIONs) have a high potential for use in biomedical applications, including controlled drug delivery, chemotherapy, drug formulations, hyperthermia treatment, and radioimmunotherapy [165]. Magnetometry techniques such as VSM (vibrating sample magnetometer) [103,176,185] and SQUID (superconducting quantum interference device) magnetometry [212] are used to determine the net magnetization of nanoparticles.

### 3.1. UV–VIS Spectroscopy

UV–vis spectroscopy, which is based on the Beer–Lambert law, is a common method for investigating the optical and structural properties of nanomaterials, such as shape, size, agglomeration state, concentration, refractive index, and colloidal stability [213,214,215,216,217]. This technique, as previously stated, can be used to confirm the formation of IONPs. Due to their surface plasmon resonance (the resonance of the metal’s conduction electrons), metal nanoparticles with a diameter of less than 100 nm efficiently scatter optical light; however, as the particle size increases, the absorption spectra can change, shifting toward longer wavelengths. The nanoparticle’s composition, environment, form, and size affect the plasmon resonance’s bandwidth, magnitude, and wavelength peak [218]. Greenly synthesized iron oxide nanoparticles typically have absorption spectra with peaks in the 290–370 nm region and an estimated energy band gap from 2.9 to 4.22 eV [106,219,220,221].

### 3.2. TEM and HRTEM

Transmission electron microscopy (TEM) is a characterization technique used to study the dynamics of physical and chemical processes of the nanostructure at an atomic resolution [222]. An electron beam interacts with the sample, and the transmitted electrons are used to form the image by magnifying and focusing them using an objective lens. The interaction of the electron beam with the sample in the TEM image results in diffraction processes as opposed to absorption processes, which occur in the light microscopic image. Because the sample will be exposed to a high vacuum during the analysis, it must be prepared to endure both the electron beam and the high-vacuum chamber [223]. The difference in electron densities of the structures, the density and thickness of the sample, the objective aperture’s diameter, and the energy of the electrons all influence TEM images [224]. Additionally, the contrast image can be generated using either unscattered electrons (light field image) or deflected electrons (dark field image). This technique is widely used to characterize the morphology of nanostructures, such as particle size [225,226], agglomeration state [134], electronic state, and chemical composition [227]. Furthermore, depending on the sample, the electron energies for the beam are generally 100 keV for conventional images and 1 MeV for high-resolution imaging [223]. High-resolution transmission electron microscopy (HRTEM) is a TEM imaging mode that provides atomic-level imaging of a nanoparticle’s crystalline structure. With this level of detail, it is feasible to image crystal structures, crystal defects, and individual atoms [218]. 

### 3.3. SEM

Scanning electron microscopy (SEM) is a non-destructive analytical method used for surface and microstructural analysis that also uses an electron beam, typically ranging from 1 to 30 keV, that scans the surface of the sample [223,228]. This technique is also useful for determining the purity, homogeneity, and degree of dispersion [229]. When the electron beam strikes a nanoparticle, it emits X-rays as well as three types of electrons: backscattered (or primary) electrons, secondary electrons, and Auger electrons. SEM makes use of both primary and secondary electrons, with the latter being used to generate high-resolution images that reveal features as small as 1–5 nm [229]. An advantage of SEM characterization is that it also helps to study the surface characteristics of the sample of IONPs, given the three-dimensional appearance of the surface structure [127]. 

Nanoparticles do not require pretreatment since metals conduct electricity when struck by an electron beam, whereas non-metallic materials do. To do this, they are often coated with gold to create a thin coating of conductive material. In standard SEM, however, water molecules evaporate in a vacuum, producing an impediment for the electron beams and obscuring image clarity; hence, water removal is important. 

### 3.4. EDX

Energy-dispersive X-ray spectroscopy (EDX) is a technique for determining the purity and elemental composition of synthesized nanoparticles [230]. As previously stated, when an electron beam hits a nanoparticle, X-rays are released along with the three types of electrons, and the energy of the generated X-rays depends on the material under investigation; hence, EDX is a technique used in combination with SEM. An image of each element in the sample may be obtained by moving the electron beam over the material, giving an overall mapping of the sample by examining near- and at-the-surface elements and estimating its proportion at different points [218].

### 3.5. XRD

X-ray diffraction (XRD) is a non-destructive technique used to analyze the crystallographic structure of a material. It can also be used to determine element proportions, the position of atoms in the unit cell, unit cell characteristics, and the number of atoms in the unit cell [231]. The interaction of the X-ray beam with the atomic planes results in partial beam transmission, with the remainder absorbed, refracted, scattered, and diffracted by the sample [218]. High-resolution powder X-ray diffraction (HRXRD) can be used to obtain finer information about the structure of nanoparticles, such as ion spacing and bond length of related substances in single crystal cells, and it is one of the most widely used techniques for determining crystalline perfection and defect studies in single crystals [232], as well as detailed studies of structure and phase transformation in materials [233].

The lattice and structural characteristics may be determined by measuring the diffraction angle obtained when an X-ray beam is incident on the nanoparticles. For example, the crystal size may be determined by applying the original Scherrer equation or a modified version (1)

L = k/βcos θ or ln β = ln k/L + ln 1/cos θ(1)

where L = average crystallite size, k = constant related to shape (0.94), λ = wavelength of X-ray, β = peak width of the diffraction peak profile at half maximum height, and θ = Bragg’s angle [234].

### 3.6. DLS

Dynamic light scattering (DLS), also known as photon correlation spectroscopy or quasi-elastic light scattering, is a frequently employed technique for determining the size distribution profile of NPs in colloidal solutions in the nano- and submicrometer ranges [163]. When a monochromatic laser beam strikes a suspension of tiny particles, the light scatters in all directions, depending on the size and shape of the nanoparticles [235]. Given that the nanoparticles in suspension are in continuous motion due to Brownian motion, the scattering intensity changes over time and offers information on particle size, allowing the hydrodynamic diameter of NPs in solution to be determined. The hydrodynamic diameter is the diameter of the NPs and solvent molecules that diffuse at the same rate as the colloid [236,237]. DLS can also measure the stability of nanoparticles over time, which is useful for understanding how they could behave in different environments and applications [238].

### 3.7. SQUID

As previously stated, the magnetic properties of nanoparticles can be studied using a superconducting quantum interference device (SQUID), a very sensitive magnetometer based on superconducting loops incorporating Josephson junctions, which converts the magnetic flux into an electric current having an extremely low magnetic flux noise [239]. This technique is sensitive enough to measure fields as low as 10^−18^ T, and allows for the measurement of various magnetic properties such as magnetization saturation (M_S_), magnetization remanence (M_R_), and blocking temperature (TB) [240,241]. The development of nanoSQUID has also allowed the measurement of the magnetic response of individual molecules [242,243], which provides the advantage of direct measurement of magnetization changes in small spin systems. Another method used for the same purpose is SQUID-on-tip (SOT), which has single-electron spin sensitivity and can detect high-field and ultra-low temperature nanomagnetic imaging straight from a single nanoparticle [244].

### 3.8. VSM

A vibrating-sample magnetometer (VSM) (also referred to as a Foner magnetometer) is another method that can be used to measure magnetic properties based on Faraday’s Law of Induction. Unlike the squid, VSM does not need superconductors or cryogenics [163,245]; therefore, it is more widely used.

In a constant magnetic field, a nanoparticle will magnetize and align with the external field, causing its magnetic dipole moment to generate a magnetic field around it (magnetic stray field). Moving the sample up and down changes the magnetic stray field as a function of time, inducing an electric field in the pick-up coils proportional to the nanoparticle’s magnetization according to Faraday’s Law of Induction [246]. This technique also allows for the determination of magnetization saturation and magnetization remanence [247,248] and they are studied as a function of magnetic field, temperature, and time [249].

### 3.9. Zeta Potential (ζ)

Since the zeta potential cannot be measured directly, unlike the other parameters described above, all the equipment used for determining its value measures another parameter, such as the electrophoretic mobility and the colloid vibration current, then it uses those measurements to calculate the zeta potential. Electrophoretic and electroacoustic methods are commonly used to measure zeta potential [250]. 

The first method is based on electrophoresis, which is based on the movement of particles in an electric field. This movement occurs because the nanoparticles interact with the field due to electrical charges on their surface. The electric field, surface charge of the nanoparticles, and suspension medium determine the direction and velocity of the movement [218]. Electrophoretic light scattering (ELS) is used through a laser Doppler arrangement to measure the velocity of the nanoparticles. This enables the determination of the moving particles’ velocity and direction within the measurement cell’s frame of reference [250].

On the other hand, electroacoustics methods can be described as the study of electrokinetics at high frequency. It results from the interaction between acoustic and electric fields when ultrasound waves propagate through a fluid medium containing ions [251,252]. There are two types of electroacoustic processes based on the driving force that produces particle movement in one direction relative to the liquid: colloid vibration potential (CVP) or colloid vibration current (CVI) and electrokinetic sonic amplitude (ESA) [252]. In CVP, an AC electric current is generated by charged colloidal particles moving relative to the liquid under the influence of an ultrasound wave [250]. The opposite effect occurs in ESA because it utilizes an AC electric field that oscillates nanoparticles in the liquid. Each particle’s oscillation generates small acoustic dipoles, which generate an ultrasonic wave that can be detected by a transducer as a function of the applied frequency [253,254].

## 4. Antimicrobial Properties of Green-Mediated Synthesis Iron Oxide Nanoparticles

### 4.1. Antibacterial Activity

Considering the emergence of antibiotic-resistant strains as the leading public health threat worldwide, the search for novel molecules with antibacterial potential is of particular interest. Free iron ions and various metal and metal oxide NPs like silver, ZnO, Al_2_O_3_, and TiO_2_ have been screened as bactericidal or antibacterial agents [255,256]. However, the main concern when using nanoparticles with antibacterial properties is their high toxicity, which has been reported for ZnO [63,64]; thus, a nanoparticle is needed that is biologically active against bacteria and biocompatible [61,62]. In this context, IONPs have recently emerged as an alternative due to their bactericide properties and biocompatibility in vivo and in vitro. Despite the potential of nanoparticles as antimicrobial agents, some risks have been reported concerning human health and the environment. In contrast to this, IONPs have been shown to be safe and do not cause toxicity in mammalian cell cultures [257,258], a highly desired characteristic to use in biomedical, clinical, and pharmaceutical applications. The most relevant IONPs with biological activity are Fe_2_O_3_, magnetite Fe_3_O_4_, and limonite Fe_2_O_3_·H_2_O [259,260].

#### 4.1.1. Mechanisms of Antibacterial IONPs Activity

Typically, the antibacterial efficacy of nanoparticles is derived from several mechanisms concerning the interaction between the nanoparticles and bacterial cells [261]. The specific mode of action can be contingent upon the distinct attributes of the nanoparticles and the bacterial strains in question. The process entails the adherence of nanoparticles (NPs) to the bacterial surface, which subsequently penetrate the bacterial cell attributed to their small dimensions [262]. Silver nanoparticles (AgNPs) have the capability to compromise the integrity of the bacterial membrane by forming holes. Additionally, AgNPs can engage with intracellular proteins, leading to the production of reactive oxygen species (ROS), inducing oxidative stress and cellular impairment [8,261].

##### Attachment to the Cell Membrane

The attachment of IONPs to the bacterial cell membrane initiates a series of events that compromise the integrity and functionality of the membrane, leading to bacterial cell death [263]. The antimicrobial action of IONPs is multifaceted and can involve a combination of mechanisms, depending on the specific conditions and bacterial strains involved. This results in the obstruction of the external cell barriers and impedes transport functionalities. When IONPs adhere to the bacterial cell membrane, they can cause physical disruption due to their nano-sized nature and high surface area to volume ratio. The nanoparticles can create localized stress points or disturbances on the membrane, which may lead to structural deformities or mechanical damage [264]. 

The attachment and subsequent interaction of IONPs with bacterial cell membranes can alter their permeability [265]. This can lead to leakage of vital intracellular contents, like ions, nutrients, and other small molecules. An altered permeability can disturb the cellular homeostasis, which is critical for bacterial survival.

##### Membrane Disruption

As mentioned, iron oxide nanoparticles can interact with bacterial cell membranes via electrostatic interactions, producing harmful oxidative stress in the bacterium by generating free radicals [266]. Additionally, intracellular oxygen in aerobic bacteria oxidizes zero-valent iron, causing irreversible oxidative damage to the cell. Moreover, because of their small size, IONPs can enter the cell and cause physical damage to the cell wall, limiting cellular growth, replication, and even communication [267].

In *E. coli*, electron microscope analysis revealed that Fe_2_O_3_ nanoparticles bind to the cell wall and even penetrate into the cytoplasm, where they accumulate, causing vacuole formation and cell wall disruption [268,269]. Fe_3_O_4_ nanoparticles bind to the formate hydrogenlyase complex (FHL) found in Gram-negative bacteria cell walls, generating a gradient in the inner membrane that has a strong antimicrobial effect on these bacteria [270]. Another mechanism that causes membrane disruption is disturbing the activity of F0/F1-ATPase, which reduces the membrane redox potential and, as a result, the H^+^ rate [270]. The size and shape of IONPs have been suggested as key factors in elucidating their bactericidal activity [271].

ROS damage DNA molecules through their genotoxic action [272]. A decrease in the activity of antioxidant system enzymes (SOD, catalase, and glutathione reductase) might cause an increase in ROS concentration [67]. Metal ions are able to bind the mercapto (-SH), amino (–NH), and carboxyl (–COOH) groups of proteins, including enzymes, causing inactivation or partial inhibition [271]. IONPs can reduce the expression of antibiotic resistance genes (ARGs) in antibiotic-resistant bacteria found in operating rooms [259]. The capacity of nanoparticles with small sizes to inhibit DNA replication by inactivating topoisomerase has also been reported [273]. ROS interacts with thiol groups in membrane proteins, generating oxidative stress, which denatures the protein, resulting in membrane impermeability and, finally, microbial death [67].

##### Enzyme Denaturation

Iron oxide nanoparticles (IONPs) have shown promising antibacterial activity, partly due to their ability to denature enzymes as well as inhibit enzyme activity [25]. For example, IONPs can directly bind to enzymes, altering their three-dimensional structure and thus their functionality [274]. The nanoparticles may attach to active sites or other critical regions of the enzyme, hindering substrate binding and catalytic activity [275]. Also, the interaction between IONPs and enzymes may induce conformational changes in the enzyme structure. Such alterations can render the enzyme inactive or significantly reduce its activity [23]. Regarding the enzyme activity inhibition, IONPs might act as competitive inhibitors for certain enzymes, binding to their active sites and preventing substrate molecules from accessing these sites, thus inhibiting enzyme activity [276]. IONPs could also bind to sites on the enzyme other than the active site, altering the enzyme’s shape and reducing its activity, a form of non-competitive inhibition [277].

##### Electron Transfer Disruption

Electron transfer is vital for many cellular processes, especially in energy production and maintaining cellular redox homeostasis. Disruption of electron transfer can lead to cellular dysfunction and death. Iron oxide nanoparticles (IONPs) can interrupt electron transfer in several ways including interacting directly with redox-active proteins, especially those present in the electron transport chain (ETC) of bacteria [8]. This could result in the blockade or misdirection of electron flow, leading to the malfunction of the ETC and reduced ATP production. IONPs might undergo redox cycling with cellular components like NADH or flavins [278]. This cycling can drain electrons from these cellular components, disrupting the balance of oxidized and reduced forms. This can further interfere with the energy-producing and biosynthetic pathways of the cell [279,280].

#### 4.1.2. Antibiofilm Activities

The physicochemical properties of nanoparticles, such as surface charge and hydrophobicity, are important when evaluating their antibiofilm activity because they influence their capacity to adsorb and penetrate biofilms [281,282]. Positively charged and neutral Fe_3_O_4_ nanoparticles have been shown to reduce biofilm formation in *Streptococcus mutans*, the primary cause of caries, as compared to negatively charged IONPs [283]. It has also been observed that IONPs decreases alginate production, which is a key component of the exopolysaccharide of *P. aeruginosa* biofilm. This reduction is dose-dependent at concentrations ranging from 0.8 to 64 μg/mL [284]. Superparamagnetic iron oxide nanoparticles (SPIONs) cause cell death and biofilm destruction due to vibration damage, local hyperthermia, and ROS generation [285]. These factors lead to the dissociation of bacteria from a biofilm, damage to the bacterial cell wall, membrane rupture, the fusion of different cells with each other, and death [286]. A list of relevant bacteria in the medical field that are susceptible to IONPs is presented in Table 7.

Some literature on IONPs’ antibacterial activity often reports the minimum inhibitory concentration (MIC) values ranging from a few micrograms per milliliter (µg/mL) to several hundred µg/mL as summarized next:

- Low MIC values: below 50 µg/mL—this suggests a high antibacterial activity [297,298,299];
- Intermediate MIC values: 50–200 µg/mL [265,300,301];
- High MIC values: above 200 µg/mL—indicates that a higher concentration of IONPs is required for antibacterial action, suggesting lower efficacy [284,302,303].

It is important to highlight that MIC values for iron oxide nanoparticles (IONPs) with antibacterial activity can vary based on several factors such as type of bacteria, size of IONPs, synthesis method, and experimental conditions [299].

### 4.2. Antifungal Activity

Iron oxide nanoparticles are a promising and safe antifungal agent with many applications, including medicinal, agricultural, and environmental applications [304]. Due to the benefits of sustainability, environmental considerations, and less toxic waste production, the green synthesis of nanoparticles used for biomedical applications is gaining popularity over chemically synthesized agents. Research on the antifungal activity of IONPs derived from green synthesis is still limited, although results against a wide range of pathogenic fungi are promising [305] (see also Table 8). 

For instance, IONPs (mixed Fe_2_O_3_ and Fe_3_O_4_) synthesized using tannic acid in a green method showed antimycotic activity against *Trichothecium roseum*, *Cladosporium herbarum*, *Penicillium chrysogenum*, *Alternaria alternata*, *and Aspergillus niger* [305]. The antifungal activity was measured by inhibiting spore germination and determining the zone of inhibition (agar well diffusion assay) of fungal pathogens using a concentration of 0.5 mg/mL in both assays, with an inhibition range of 87.74–70.67% and an inhibition zone in the range of 28.67–18.00 mm (Table 8). The minimum inhibitory concentration (MIC) varied between 0.063 mg/mL and 0.016 mg/mL for the most sensitive pathogens, *Penicillium chrysogenum*, and *Aspergillus niger*, and was comparable with the MIC value shown by the conventional fungicide hexaconazole, which was used as a standard control [305]. 

Mixed α-Fe_2_O_3_ and γ-Fe_2_O_3_ IONPs synthesized from Fe(NO_3_)_3_·9H_2_O, using *Platanus orientalis* leaf extract in an aqueous phase green synthesis approach, also showed promising antifungal activity against *Aspergillus niger* and even stronger activity against *Mucor piriformis*, with inhibition zones in the agar well diffusion assay of 16 mm and 26 mm, respectively, using 75 μL of 0.10 mg/mL NPs. These inhibition zones were also equivalent to hexaconazole (Table 8) [17]. The authors attributed the high activity against *Mucor piriformi* to the fungus’s stronger surface interaction with the IONPs.

IONPs generated from FeCl_3_·6H_2_O with green and black tea leaf extracts were tested against *Aspergillus flavus* and *Aspergillus parasiticus* strains and compared with the antifungal activity of copper and silver nanoparticles. Furthermore, their effects on aflatoxin production and adsorption were also evaluated. While silver nanoparticles had greater antimicrobial activity, IONPs displayed the highest adsorption capabilities of aflatoxin B1, giving them an advantage in applications such as food safety. A concentration of 100 µg/mL IONPs reduced the aflatoxin production by 39.2–43.5% in Aspergillus flavus and 47.1–51.6% in *Aspergillus parasiticus*, suggesting significant inhibition of the fungal pathogens (Table 8) [306]. 

Several other studies have been performed using a variety of biological source materials and green synthesis approaches for IONPs and tested against various fungal strains, which are summarized in Table 8. Briefly, Laurus nobilis L. extracts for IONP generation results in antifungal activity against *Aspergillus flavus* and *Penicillium spinulosum* [3]; leaf extract of *Euphorbia helioscopia* aids IONP generation with inhibitory activity against *Cladosporium herbarum* [307]; and *Euphorbia hirta* leaf extract can be used to generate IONPs with antifungal activity against *Aspergillus fumigatus*, *Aspergillus niger*, and *Arthogrophis cuboida* (Table 8) [121]. 

It is worth noting that nanoparticles generally allow for surface modification to either change the physicochemical properties or even for targeted drug or bio-cargo delivery via the coating/adsorption/binding of therapeutic and bioactive molecules. One such example is chitosan-coated IONPs with antimycotic activity against *Fusarium solani*, *Aspergillus niger*, and *Candida albicans* [308]. While these IONPs were not synthesized in a green approach, the same method used for their modification should be applicable to IONPs from green synthesis. Labeling IONPs from green synthesis with approved fungicidal drugs should also be viable, allowing for more targeted delivery with dual antifungal effects. However, studies using this strategy are currently insufficient, and the safety of such modified IONPs will need to be carefully investigated [309]. 

The mechanism of action of IONPs for their antifungal activity has not been investigated in detail; however, it is expected to involve the same mechanisms that are responsible for their antibacterial activity. This includes the effects on cell membrane permeability and the generation of radical oxygen species (ROS) via Fenton reactions or photocatalytic reactions with dissolved oxygen, which cause damage to the cell’s DNA, cell wall components, and proteins, resulting in cell growth inhibition and cell death. The nanoparticles’ high surface-to-volume ratio allows them to strongly adhere to the cell surface of fungi, while their small size allows them to penetrate and damage the cell wall, directing their effects toward the microbial cells [17]. To fully understand the mechanism of action for the antimycotic activity of IONPs derived from green synthesis, more research will be required, which will also help us better understand the different activity profiles of varying IONPs against different fungal strains.

A summary of the described studies is provided in Table 8 and reveals that the antimicrobial activity of IONPs generated by green synthesis can vary depending on the biological source material and synthesis approach used.

### 4.3. Antiparasitic Activity

IONPs derived either from green or chemical synthesis have been used as antiplasmodial and antileishmanial agents; however, most studies have used chemically synthesized NPs [310,311], and only few reports exist of the use of green synthesized IONPs (Table 9). Furthermore, the IONPs have been used in combination with known antiparasitic drugs (i.e., artesunate, albendazole) to enhance their inhibitory effect [311,312,313]. Nevertheless, to the best of our knowledge, none of the studies have used green synthesized IONPs. A key feature is that IONPs have not shown cytotoxic effects in vitro, making them attractive for pharmaceutical and medical applications [31].

**Figure 2:**
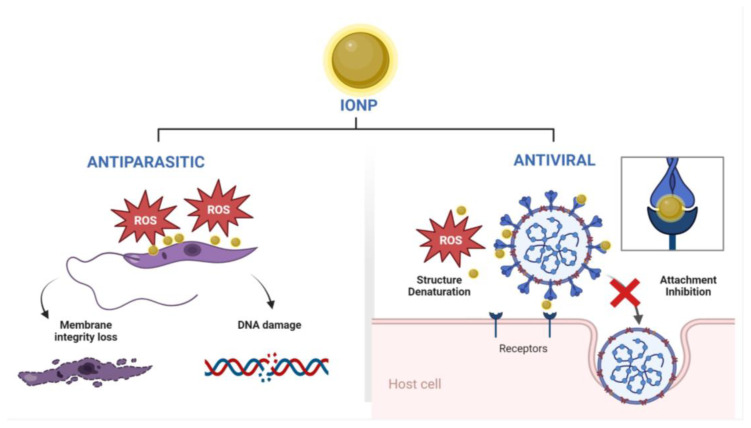
Schematic representation of proposed mechanisms of antiparasitic and antiviral activity mediated by IONPs. Figure created with BioRender.com.

When the antileishmanial activity (IC_50_) of chemically synthesized IONPs was compared to that of biologically synthesized IONPs from *Trigonella foenum-graecum* plant extract, no greater difference against both promastigotes and amastigotes of *Leishmania tropica* was observed, despite the fact that the IC_50_s of biologically synthesized IONPs were slightly lower. However, an enhanced inhibitory effect was observed under LED exposure [314]. High levels of reactive oxygen species were found to be responsible for parasite DNA damage and membrane integrity loss (Figure 2). Notably, these green synthesized IONPs proved to be biocompatible with human erythrocytes. Overall, the results showed that biologically produced IONPs had promising antiprotozoal agent capabilities [314]. In another study, IONPs synthesized from FeCl_3_·6H_2_O and *Nephrolepis exaltata* aqueous extract showed favorable parasite inhibitory activity against Plasmodium at 25 μg/mL (62% inhibition) when compared to chloroquine control samples (70% inhibition) at the same concentration [31]; however, the mechanism of action was not described.

In general, these IONPs show encouraging antiparasitic properties. Nevertheless, most studies (although limited) are based on non-green synthesized IONPs. As a result, further research should be carried out using IONPs obtained through green synthesis to better understand their potential to inhibit parasite growth.

### 4.4. Antiviral Activity

Nanotechnology and its applications have shown great potential in the development of novel diagnostic and therapeutic approaches against viral infections [316]. In particular, the literature has reported exciting discoveries regarding the antiviral activity of metal complexes and metal-based nanomaterials [317]. Most of these studies involve both the use of the nanoparticles alone or the coating of nanoparticle surfaces with known antiviral compounds [318,319]. The main antiviral mechanisms observed in metal and metal oxide nanoparticles are the inhibition of viral attachment and uncoating by viral-attachment-protein hijacking, disruption of glycoprotein disulfide bonds, and viral structure denaturation via oxidation (reactive oxygen species or ROS) (Figure 2) [320]. However, the study of iron oxide nanoparticles (IONPs) has received little attention as potential antivirals. Chemically synthesized IONPs had antiviral activity against the H1N1 Influenza A virus [321,322]. The use of approximately 2 pg of IONPs reduced the viral NP transcript by about 100-fold [322]. However, the human and environmental effects of the inherent toxicity associated with laboratory chemical synthesis and its byproducts are a serious issue [323]. A recent study described a moderate inhibition of polio virus-1 and polio virus-2 after treatment of infected L20B (mice cell line transfected with poliovirus receptor) and RD (human rhabdomyosarcoma) cells with IONPs synthesized from the aqueous fruit extracts of *Hyphaene thebaica* (Table 9) [315]. Overall, the antiviral potential of IONPs is vast and still remains unexplored, which poses a fundamental question to be fulfilled by future studies regarding green-synthesized nanoparticles.

## 5. Conclusions and Future Prospects

The present review provides a thorough examination of iron oxide nanoparticles (IONPs), specifically focusing on their synthesis through environmentally sustainable methods, their notable antimicrobial capabilities, and the significance of the most prominent characterization methods. This combination of factors sheds light on both the potential benefits and challenges associated with the utilization of IONPs as antimicrobial agents.

The green synthesis of IONPs, frequently involving the utilization of plant extracts, microorganisms, or biocompatible materials, aligns cohesively with the contemporary emphasis on sustainability and eco-friendly practices within the realm of nanotechnology. Such an environmentally conscious approach not only minimizes the ecological footprint but also engenders the development of nanomaterials that are safer and more biocompatible.

The demonstrated antimicrobial efficacy of IONPs, spanning a broad spectrum of microorganisms encompassing bacteria, fungi, parasites, and viruses, represents a promising avenue for addressing the escalating global challenge of antimicrobial resistance. The observed synergistic effects when IONPs are combined with traditional antibiotics present a potential solution for countering resistant strains. 

While there are shared mechanisms like ROS generation, the unique structures and life cycles of bacteria, fungi, parasites, and viruses mean that IONPs might interact differently. For example, whereas ROS generation and cell wall interaction are mechanisms for the IONP’s antimicrobial activity both in bacteria and fungi, in the latter organisms, the IONPs could interfere with the electron transport chain in fungal mitochondria, inhibiting ATP production, which is a mechanism particular to eukaryotes. On the other hand, a particular mechanism in the case of viruses is that IONPs can interfere with the assembly of new viral particles or their release from host cells. It is also worth noting that the effectiveness of IONPs can vary depending on their size, shape, coating, and concentration. Further studies are necessary to fully elucidate the specific mechanisms of IONPs against different microorganisms.

Nevertheless, it is imperative to acknowledge and confront the constraints and apprehensions surrounding the use of IONPs. Cytotoxicity remains a substantial challenge, necessitating meticulous optimization of dosage, surface functionalization, and comprehensive assessments of biocompatibility. Furthermore, the long-term consequences of IONP exposure and the prospect of their accumulation in biological systems demand further rigorous investigation.

Looking forward, the prospects of IONPs as antimicrobial agents appear promising. Advancements in surface modification techniques, innovative strategies for targeted delivery, and rigorous regulatory approval procedures are poised to unlock their full potential. Additionally, mindful consideration of their potential environmental implications and the implementation of responsible disposal methodologies are integral components of future research and applications.

In essence, the combination of green synthesis methodologies, potent antimicrobial attributes, and the rigorous characterization of IONPs has unveiled a promising trajectory for combating microbial infections. While challenges persist, the convergence of research endeavors, accompanied by an unwavering commitment to safety and sustainability, offers a glimpse into a future where IONPs stand poised to revolutionize the landscape of antimicrobial therapeutics, thereby mitigating the escalating global threat of antimicrobial resistance.

## Figures and Tables

**Table 1:** Iron oxide nanoparticles synthesized by bacterial species.

Bacterial Strain	Type of Nanoparticle	Mechanism of Synthesis	Size of Nanoparticle (nm)	Nanoparticle Morphology	Ref.
*Alcaligens faecalis*	Fe_2_O_3_	Extracellular	12.3	Irregular spherical	[43]
*Bacillus cereus*	Fe_3_O_4_	Extracellular	29.3	Spherical	[29]
*Bacillus megaterium*	FeO-NPs	-	20–30	-	[44]
*Bacillus subtilis*	Fe_3_O_4_	Extracellular	60–80	Spherical	[41]
*Bacillus subtilis* *Bacillus pasteurii* *Bacillus licheniformis*	Fe_3_O_4_	Extracellular	37–97	Rhombohedral	[45]
*Escherichia coli*	Fe_3_O_4_	Extracellular	23 ± 1	Spherical	[46]
*Geobacter sulfurreducens*	Fe_3_O_4_	Extracellular	10–50	-	[47]
*Gluconacetobacter xylinus*	Fe_3_O_4_	Intracellular	50	-	[48]
*Lactobacillus casei*	Fe_3_O_4_	Intracellular	10–15	Spherical	[42]
*Lactobacillus fermentum*	Fe_3_O_4_	Intracellular	10–15	Spherical	[39]
*Magnetospirillum magneticum*	Fe_3_O_4_	Intracellular	10–60	Cuboidal, rectangular, and spherical NPs	[49]
*Magnetospirillum gryphiswaldense*	Fe_3_O_4_	Extracellular/intracellular	25–55	Polydisperse	[50]
*Microbacterium marinilacus*	FeO-NPs	Extracellular	25–88	Irregular	[51]
*Nitrospirae (MYR-1)*	Fe_2_O_3_	Intracellular	40	Bullet shaped	[52]
*Pseudomonas stutzeri*	Fe_2_O_3_	-	10–20	-	[53]
*Paenibacillus polymyxa*	FeO-NPs	-	26.65	Spherical	[54]
*Proteus mirabilis*	Fe_3_O_4_	Intracellular	1.44–1.92	Spherical	[55]
*Pseudomonas aeruginosa*	Fe_3_O_4_	Extracellular	23	Spherical	[46]
*Streptococcus suis*	Fe_3_O_4_	-	-	-	[56]
*Thiobacillus thioparus*	Fe_3_O_4_	Intracellular	-	-	[57]

**Table 2:** Synthesis of iron oxide nanoparticles using fungi and yeast.

Fungi	Location of Synthesis	Type of Nanoparticle	Size of Nanoparticle (nm)	Nanoparticle Morphology	Ref.
*Alternaria alternate*	Cell-free filtrate	γ-Fe_2_O_3_	75–650	Cubic	[71]
*Aspergillus fumigatus*	Fungal biomass	Fe_2_O_3_	42.4	Irregular spherical	[67]
*Aspergillus japonicus*	Fungal biomass	Fe_3_O_4_	60–70	Cubic	[68]
*Aspergillus niger YESM 1*	Fungal homogenate	Fe_3_O_4_	18–50	Spherical	[72]
*Aspergillus niger BSC-1*	Cell-free filtrate	Fe_3_O_4_	20–40	Orthorhombic	[58]
*Aspergillus wentii*	Fungal biomass	Fe_2_O_3_	46	-	[67]
Baker’s yeast	-	Fe_2_O_3_	2–10	-	[73]
*Candida bombicola*	-	Fe_3_O_4_	8.5–4.5	-	[70]
*Chaetomium globosum*	Fungal biomass	Fe_2_O_3_	25.3	Irregular spherical	[67]
*Cryptococcus humicola*	-	Fe_3_O_4_	8–9	Spherical	[74]
*Curvularia lunata*	Fungal biomass	Fe_2_O_3_	20.8	Irregular spherical	[67]
*Daedalea* mushroom	Biomass	FeO NPs	16.8	Irregular shape	[33]
*Fusarium incarnatum*	Fungal cell filtrate	Fe_3_O_4_	30.56 ± 8.68	Spherical	[69]
*Neurospora crassa*	Fungal biomass	Fe_3_O_4_	50	Coralline appearance	[75]
*Phialemoniopsis ocularis*	Fungal cell filtrate	Fe_3_O_4_	13.13 ± 4.32	Spherical	[69]
*Pochonia chlamydosporium*	Fungal biomass	Fe_2_O_3_	~12–50	Spherical	[67]
*Thichoderma asperellum*	Fungal cell filtrate	Fe_3_O_4_	25 ± 3.94	Spherical	[69]

**Table 3:** Synthesis of iron oxide nanoparticles using algae.

Algae	Location of Synthesis	Type of Nanoparticle	Size of Nanoparticle (nm)	Nanoparticle Morphology	Ref.
*Chaetomorpha antennina*	Cell-free extract	Fe_3_O_4_	9–10	-	[84]
*Chlorella K01*	Dried powder	Fe_3_O_4_	76.5	Spherical	[18]
*Chlorella vulgaris*	Cell extract	FeO NPs	109	Cylindrical spheres	[10]
*Colpomenia sinuosa*	Dried powder	Fe_3_O_4_	11.24–33.71	Spherical	[81]
*Colpomenia sinuosa*	Dried powder	Fe_3_O_4_	17.78	Cubic	[85]
*Kappaphycus alvarezzi*	Dried powder	Fe_3_O_4_	14.7	Spherical	[86,87]
*Leptolyngbya* sp. *L-2*	Cell extract	FeO NPs	23	Spherical	[88]
*Oscillatoria limnetica*	Dried powder	Fe_2_O_3_	23.33	Trigonal rhombohedral	[89]
*Padina pavonica*	Dried powder	Fe_3_O_4_	31.41	Spherical	[85]
*Padina pavonica*	Freeze-dried powder	Fe_3_O_4_	10–19.5	Spherical	[90]
*Petalonia fascia*	Dried powder	Fe_3_O_4_	9.42	Spherical	[85]
*Pterocladia capillacea*	Dried powder	Fe_3_O_4_	16.85–22.47	Spherical	[81]
*Sargassum acinarium*	Freeze-dried powder	Fe_3_O_4_	21.6–27.4	Spherical	[90]
*Sargassum muticum*	Freeze-dried powder	Fe_3_O_4_	18	Cubic	[78]
*Spatoglossum asperum*	Cell extract	Fe_3_O_4_	16	Spherical	[91]
*Spirogyra hyalina*	Dried powder	Fe_3_O_4_	52	Spherical	[92]
*Spirulina platensis*	Dried powder	Fe_3_O_4_	10	-	[93]
*Turbinaria turbinata*	Cell-free extract	Fe_3_O_4_	8–14	-	[84]

**Table 4:** Synthesis of iron oxide nanoparticles using plants.

Plant Species	Part of the Plant	Type of Nanoparticle	Size of Nanoparticle (nm)	Nanoparticle Morphology	Ref.
*Aloe barbadensis*	Leaves	Fe_2_O_3_	80–100	Spherical	[100]
*Ajuga bracteosa*	Plant	Fe_3_O_4_	75	Rod shaped	[92]
Amla	Seeds	Fe_2_O_3_	2–5	Spherical	[101]
*Anastatica hierochuntica*	Plant	FeO-NPs	30–70	Spherical	[102]
*Arisaema amurense*	Root	Fe_2_O_3_	24.55 ± 6.9	Nearly spherical	[103]
*Artemisia annua*	Leaves	Fe_2_O_3_/Fe_3_O_4_	4.7 ± 0.8	Spherical	[104]
*Aspalathus linearis*	Leaves	α-Fe_2_O_3_	3–31	Spherical	[105]
*Avecinnia marina*	Flowers	FeO-NPs	10–40	Non-uniform	[106]
*Azadirachta indica*	Leaves	Fe_3_O_4_	9–12	Irregular	[107]
*Bombax malabaricum*	Leaves	Fe3O4	9	-	[108]
*Caesalpinia coriaria*	Fruit	α-Fe_2_O_3_	-	Irregular	[109]
*Calliandra haematocephala*	Leaves	Fe_3_O_4_	85.4–87.9	Bead-like spherical	[110]
*Carica papaya*	Leaves	α-Fe_2_O_3_	2.15	Spherical to agglomerate	[19]
*Centaurea solstitialis*	Leaves	FeO-NPs	-	Spherical	[111]
*Coriandrum sativum*	Leaves	FeO-NPs	20–90	Spherical	[112]
*Colocasia esculenta*	Leaves	FeO-NPs	15	Spherical	[113]
*Cynometra ramiflora*	Leaves	FeO-NPs	-	Agglomerated	[114]
*Cynara cardunculus*	Leaves	Fe_3_O_4_	13.5	Semi-spherical	[115]
*Daphne mezereum*	Leaves	Fe_3_O_4_	6.5–14.9	Spherical	[116]
*Eichhornia crassipes*	Leaves	FeO-NPs	>100	Rod shaped	[117]
*Eucalyptus* globulus	Leaves	β-Fe_2_O_3_	100	Spherical	[118]
*Eucalyptus* globulus	Leaves	Fe_3_O_4_	2.34 ± 0.53	Spherical	[119]
*Euphorbia wallichii*	Leaves	FeO-NPs	10–15	Spherical	[120]
*Euphorbia herita*	Leaves	Fe_3_O_4_	25–80	Spherical	[121]
*Gardenia resinifera*	Leaves	α-Fe_2_O_3_	3–8	Spherical	[122]
Grapes	Seeds	Fe_3_O_4_	35	Spherical	[123]
Green tea	Leaves	FeO-NPs	5.7 ± 4.1	Spherical	[124]
*Hibiscus rosasinensis*	Flower	FeO-NPs	65	Spinel	[125]
*Sageretia thea*	Leaves	Fe_2_O_3_	29	Tetragonal crystalline	[126]
*Lagenaria siceraria*	Leaves	Fe_3_O_4_	30–100	Cube	[127]
*Lantana camara*	Leaves	FeO-NPs	-	Crystalline nanorods	[128]
*Laurus nobilis* L.	Leaves	α-Fe_2_O_3_	8.03 ± 8.99	Spherical	[3]
*Lawsonia inermis*	Plant	FeO-NPs	150–200	Spherical	[129]
*Mangifera indica* L.	Leaves	FeO-NPs	-	Polycrystalline nanorod	[130]
*Mansoa alliacea*	Leaves	β-Fe_2_O_3_	18.22	Spherical nanoparticles	[99]
*Moringa oleifera*	Leaves	FeO-NPs	15.01 ± 6.03	Rods	[1]
*Parthenium hysterophorus*	Leaves	FeO-NPs	17.5	Spherical	[131]
*Plantago major*	Leaves	FeO-NPs	4.6–30.6	Spherical	[132]
*Platanus orientalis*	Leaves	α-Fe_2_O_3_ and γ-Fe_2_O_3_	38	Spherical	[17]
*Psidium guajava*	Leaves	FeO-NPs	1–5	Spherical	[133]
*Punica granatum*	Seeds	Fe_2_O_3_	25–55	Semi-spherical	[7]
*Rheum emodi*	Roots	α-Fe_2_O_3_	12	Spherical	[133]
*Ruellia tuberose*	Leaves	FeO-NPs	20–80	Hexagonal rods	[134]
*Rumex acetosa*	Plant	FeO-NPs	40	Amorphous	[96]
*Sesbania grandiflora*	Leaves	FeO-NPs	25–60	Agglomerated non-spherical	[135]
*Tamarix aphylla*	Leaves	Fe_2_O_3_	5–100	Oval	[136]
*Terminalia belerica*	Fruits	FeO-NPs	15–23	Spherical	[137]
*Tridax procumbens*	Leaves	Fe_3_O_4_	80–100	Irregular spherical	[138]
*Vaccinium corymbosum*	Leaves	FeO-NPs	52.4	Irregular shape non-agglomerated	[139]

**Table 5:** Iron oxide nanoparticles synthesized by biological waste.

Biological Source	Type of Waste	Size of Nanoparticle (nm)	Nanoparticle Morphology	Ref.
*Ananas comosus*	Fruit peel	10–16	Spherical	[147]
*Acacia mearnsii*	Biochar	18–35	-	[150]
*Camellia sinensis*	Tea waste	28.5	Spherical	[148]
*Citrus aurantifolia*	Fruit peel	3–10	-	[149]
*Cocos nucifera* L.	Fruit peel	10–100	Clustered	[151]
Coffee	Waste hydrochar	10–40	Spherical	[152]
*Cynometra ramiflora*	Fruit peel	10–25	Agglomerated	[153]
*Juglans regia*	Dried green husk	12.6	Cubic	[154]
Lemon	Fruit peel	3 and 10	Orthorhombic	[154]
*Malus domestica* *Citrus limon*	Fruit peel	17–25	Spherical	[155]
Orange	Fruit peel	50	Quasi spherical	[146]
Plantain	Fruit peel	30–50	Spherical	[156]
*Punica Granatum*	Fruit peel	40	Rod shaped	[157]
Rambutan	Fruit peel	100–200	Agglomerated spinel	[158]
Rice	Straw	9.9 ± 2.4	Aggregated spherical	[159]
Tangerine	Fruit peel	50	Spherical	[160]
Watermelon	Rinds	2–20	Spherical	[161]

**Table 6:** Characterization techniques for nanoparticles.

Property	Characterization Techniques
Size	TEM, XRD, DLS, NTA, SAXS, HRTEM, SEM, AFM, EXAFS, FMR, DCS, ICP-MS, UV-Vis, MALDI, NMR, TRPS, EPLS, magnetic susceptibility
Shape	TEM, HRTEM, SEM, AFM, STEM, EPLS, FMR, 3D tomography
Chemical composition	XRD, XPS, ICP-MS, ICP-OES, SEM-EDX, NMR, MFM, LEIS, EELS
Crystal structure	XRD, EXAFS, HRTEM, electron diffraction, STEM
Surface charge	Zeta potential, EPM
Magnetic properties	SQUID, VSM, Mössbauer, MFM, FMR, XMCD, magnetic susceptibility

**Table 7:** Antibacterial properties of IONPs from natural sources.

Treated Bacterial Strain	Biological Source for IONP Generation	Antibacterial Assay	Size of Nanoparticle (nm)	Nanoparticle Morphology	Inhibition Zone (mm)	Ref.
*Bacillus cereus*	*Zea mays* L. ear leaves	WDM (sinergical activity)	37.86	Spherical	9.87 ± 0.34	[287]
*Bacillus subtilis*	*Acorus calamus*/rhizome	WDM	20–30	Spherical	20 ± 4.2	[288]
	*Sida cardifolia*	WDM	10–22	Spherical	16 ± 1.0	[289]
	*Papaver somniferum*	WDM	38 ± 13	Elliptical or spherical	~7.1	[290]
*Enterobacillus* sp.	*Acorus calamus*/rhizome	WDM	20–30	Spherical	15 ± 3.1	[288]
*Escherichia coli*	*Moringa M. oleifera*	WDM	15.01 ± 6.03	Rod-like morphology	14	[1]
	Composite of *Psidium guavaja-Moringa oleifera*	WDM	82 ± 7.0	Spherical	20.1	[15]
	*Zea mays* L. leaves	WDM	37.86	Spherical	18.8 ± 0.82	[287]
	*Penicillium* sp.	DDM	11.3	Spherical	11.3 ± 0.6	[62]
	*Sida cardifolia*	WDM	10–22	Spherical	11.33 ± 0.58	[289]
	*Couroupita guianensis*	DDM	17 ± 10	Spherical	~16	[291]
	*Nigella sativa* seed extract	DDM	31.45	Spherical	12.34 ± 0.58	[292]
	*Piper betel* leaves extract	WDM	25.17	Cubic	13	[293]
	*Acorus calamus*/rhizome	WDM	20–30	Spherical	15 ± 2.5	[288]
	*Psidium guavaja*	WDM	1–6 nm	Spherical	8	[133]
	*Agrewia optiva* (AO) and *Prunus persica* (PP) leaf extract	WDM	15–60 (AO) 13–70 (PP)	Spherical	6.33 ± 0.33 (AO NPs) 6.33 ± 0.33 (PP NPs)	[294]
*Klebsiella pneumoniae*	*Penicillium* sp.	DDM	11.6	Spherical	11.3 ± 0.6	[62]
	*Agrewia optiva* (AO) and *Prunus persica* (PP) leaf extract	WDM	15–60 (AO) 13–70 (PP)	Spherical, granular	7.33 ± 0.33 (AO NPs) 6.33 ± 0.33 (PP NPs)	[294]
	*Papaver somniferum*	WDM	38 ± 13	Elliptical or spherical	~8	[290]
	*Sida cardifolia*	WDM	10–22	Spherical	12 ± 1.0	[289]
	*Couroupita guianensis*	DDM	17	Spherical	~12	[291]
*Listeria monocytogenes*	*Zea mays* L. leaves	WDM (sinergical activity)	37.86	Spherical	13.54 ± 0.30	[287]
*Pseudomona aeuruginosa*	*Penicillium* sp.	DDM	11.6	Spherical	11.3 ± 0.6	[62]
	*Moringa M. oleifera*	WDM	15.01 ± 6.03	Rod-like morphology	16	[1]
	*Agrewia optiva* (AO) and *Prunus persica* (PP) leaf extract	WDM	15–60 (AO) 13–70 (PP)	Spherical, granular	7.33 ± 0.33 (AO NPs) 10.66 ± 0.33 (PP NPs)	[294]
	*Papaver somniferum*	WDM	38 ± 13	Elliptical or spherical	~7.2	[290]
	*Withania coagulans*/Berries	DDM	15–20	Nanorods	~24	[295]
	*Aloe vera*/leaf extract	cellular damage assessed by SEM	8.26	Cubical, rhomboidal, spherical	-	[296]
	*Acorus calamus*/rhizome	WDM	20–30	Spherical	20 ± 2.4	[288]
	*Piper betel* leaves extract	WDM	25.17	Cubic	15	[293]
*Salmonella* sp.	Composite of *Psidium guavaja-Moringa oleifera*	WDM	82 ± 7.0	Spherical	24.2	[15]
	*Moringa oleifera*	WDM	15.01 ± 6.03	Rod-like morphology	18	[1]
*Shigella*	Composite of *Psidium guavaja-Moringa oleifera*	WDM	82 ± 7.0	Spherical	24.4	[15]
	*Psidium guavaja*	WDM	1–6 nm	Spherical	18	[133]
*Staphylococcus aureus*	*Penicillium* sp.	DDM	12	Spherical	12 ± 0.6	[62]
	Composite of *Psidium guavaja-Moringa oleifera*	WDM	82 ± 7.0	Spherical	26.2	[15]
	*Moringa M. oleifera*	WDM	15.01 ± 6.03	Rod-like morphology	18	[1]
	*Agrewia optiva* (AO) and *Prunus persica* (PP) leaf extract	WDM	15–60 (AO) 13–70 (PP)	Spherical, granular	12.33 ± 0.33 (AO NPs) 10.66 ± 0.33 (PP NPs)	[294]
	*Sida cardifolia*	WDM	10–22	Spherical	13.67 ± 0.58	[289]
	*Withania coagulans*/Berries	WDM	15–20	Nanorods	~23	[295]
	*Couroupita guianensis*	WDM	17	Spherical	~11	[291]
	*Nigella sativa* seed extract	WDM	31.45	Spherical	11.52 ± 0.58	[292]
	*Piper betel* leaves extract	WDM	25.17	Cubic	12	[293]
*Streptococcus mutans*	*Piper betel* leaves extract	WDM	25.17	Cubic	11	[293]
	*Agrewia optiva* (AO) and *Prunus persica* (PP) leaf extract	WDM	15–60 (AO) 13–70 (PP)	Spherical	8.66 ± 0.33 (AO NPs) 7.33 ± 0.33 (PP NPs)	[294]
*Streptococcus pyogenes*	*Zea mays* L. leaves	WDM	37.86	Spherical	13.09 ±0.15	[287]
	*Agrewia optiva* (AO) and *Prunus persica* (PP) leaf extract	WDM	15–60 (AO) 13–70 (PP)	Spherical	8.66 ± 0.33 (AO NPs) 7.33 ± 0.33 (PP NPs)	[294]

WDM: well diffusion method. DDM: disk diffusion method.

**Table 8:** Antifungal activity of iron oxide nanoparticles derived from green synthesis.

Treated Fungal Strain	Biological Source for IONP Generation	Approach	Size (nm)	Nanoparticle Morphology	Inhibition Zone (mm)	Ref.
*Alternaria alternata*	-	Tannic acid	10–30	Quasi-circular	21.33 ± 3.83	[305]
*Arthogrophis cuboida*	*Euphorbia hirta*	Leaf extract	25–80	Cavity-like structures	25.33 ± 0.5	[121]
*Aspergillus flavus*	*Laurus nobilis* L.	Leaf extract	8.03 ± 8.99	Crystalline, spherical; partly hexagonal	13	[3]
	Green tea (GT) or black tea (BT)	Leaf extract	42–60	Agglomerated and spherical	Aflatoxin reduction [%]: GT: 43.5 ± 1.5 BT: 39.2 ± 0.9	[306]
*Aspergillus fumigatus*	*Euphorbia hirta*	Leaf extract	25–80	Cavity-like structures	21.67 ± 1.5	[121]
*Aspergillus niger*	-	Tannic acid	10–30	Circular	26.33 ± 1.15	[305]
	*Platanus orientalis*	Leaf extract	38	Spherical	16	[17]
	*Euphorbia hirta*	Leaf extract	25–80	Cavity-like structures	18.67 ± 0.5	[121]
*Aspergillus parasiticus*	Green tea (GT) or black tea (BT)	Leaf extract	42–60	Agglomerated and spherical	Aflatoxin reduction [%]: GT: 51.6 ± 1.6 BT: 47.1 ± 3.1	[306]
*Cladosporium herbarum*	-	Tannic acid	10–30	Circular	18.00 ± 1.00	[305]
	*Euphorbia helioscopia*	Leaf extract	7–10	Spherical	38.33	[307]
*Mucor piriformis*	*Platanus orientalis*	Leaf extract	38	Spherical	26	[17]
*Penicillium chrysogenum*	-	Tannic acid	10–30	Circular	28.67 ± 1.53	[305]
*Penicillium spinulosum*	*Laurus nobilis* L.	Leaf extract	8.03 ± 8.99	Crystalline, spherical; partly hexagonal	14	[3]
*Trichothecium roseum*	-	Tannic acid	10–30	Circular	22.67 ± 2.52	[305]

**Table 9:** List of studies using iron oxide nanoparticles (IONPs) derived from green synthesis as antiparasitic or antiviral agents.

Treated Microorganism	Biological Source for IONP Generation	Approach	Size (nm)	Nanoparticle Morphology	Inhibitory Effect	Ref.
	Parasites					
Plasmodium	*Nephrolepis exaltata*	IONPs alone	16	Roughly spherical	62% inhibition at 25 μg/mL of IONPs	[31]
*Leishmania tropica* KWH23	*Trigonella foenum-graecum*	Synergistic effects of IONPs and LED light together	-	-	IC_50_ of 0.001572 ± 0.02 μg/mL and 0.01140 ± 0.02 μg/mL promastigotes and amastigotes	[314]
	Virus					
Poliovirus (Types 1 and 3)	*Hyphaene thebaica*	IONPs alone	10	Quasi-spherical and cuboidal		[315]

## Data Availability

All tables were created by the authors. All sources of information were adequately referenced. There was no need to obtain copyright permission.

## Abbreviations

IC_50_	The concentration of a drug or substance needed to inhibit a biological process or response by 50%
AFM	Atomic force microscope
ARGs	Antibiotic-resistance genes
ATP	Adenosine triphosphate
CFF	Cell free filtrate
DCS	Differential centrifugal sedimentation
DDM	Disk diffusion method.
DLS	Dynamic light scattering
EDX	Energy-dispersive X-ray spectroscopy
EELS	Electron energy loss spectroscopy
ETC	Electron transport chain
EPLS	Elliptically polarized light scattering
EPM	Electrophoretic mobility
EXAFS	X-ray absorption fine structure
FCF	Fungal cell filtrate
FHL	Formate hydrogenlyase complex
FMR	Ferromagnetic resonance
HRTEM	High-resolution Transmission Electron Microscopy
ICP-MS	Inductively coupled plasma mass spectrometry
ICP-OES	Inductively coupled plasma optical emission spectrometry
IONP	Iron oxide nanoparticles
LEIS	Low-energy ion scattering spectroscopy
MALDI	Matrix assisted laser desorption/ionization
MFM	Magnetic force microscopy
MIC	Minimum inhibitory concentration
NADH	Nicotinamide adenine dinucleotide
NMR	Nuclear magnetic resonance
NTA	Nanoparticle tracking analysis
NPs	Nanoparticles
ROS	Radical oxygen species
SAXS	Small Angle X-ray Scattering
SEM	Scanning electron microscopes
SOD	Superoxide dismutases
SQUID	Superconducting quantum interference device
STEM	Scanning transmission electron microscopy
TEM	Transmission electron microscopy
TRPS	Tunable resistive pulse sensing
UV-Vis	UV-visible
VSM	Vibrating sample magnetometer
WDM	Well diffusion method
XMCD	X-ray magnetic circular dichroism
XPC	X-ray photon correlation
XRD	X-ray diffraction
